# Mental Quality of Life Is Related to a Cytokine Genetic Pathway

**DOI:** 10.1371/journal.pone.0045126

**Published:** 2012-09-25

**Authors:** Dounya Schoormans, Teodora Radonic, Piet de Witte, Maarten Groenink, Donija Azim, Rene Lutter, Barbara J. M. Mulder, Mirjam A. G. Sprangers, Aeilko H. Zwinderman

**Affiliations:** 1 Department of Medical Psychology, Academic Medical Center, Amsterdam, The Netherlands; 2 Department of Cardiology, Academic Medical Center, Amsterdam, The Netherlands; 3 Interuniversity Cardiology Institute of the Netherlands, Utrecht, The Netherlands; 4 Department of Clinical Epidemiology and Biostatistics, Academic Medical Center, Amsterdam, The Netherlands; 5 Department of Radiology, Academic Medical Center, Amsterdam, The Netherlands; 6 Departments of Pulmonology and Experimental Immunology Academic Medical Center, Amsterdam, The Netherlands; South Texas Veterans Health Care System and University Health Science Center San Antonio, United States of America

## Abstract

**Background:**

Quality of life (QoL) in patients with chronic disease is impaired and cannot be solely explained by disease severity. We explored whether genetic variability and activity contributes to QoL in patients with Marfan syndrome (MFS), a genetic connective tissue disorder.

**Methodology/Principal Findings:**

In 121 MFS patients, patient characteristics (i.e. demographics and MFS-related symptoms) were assessed. Patients completed the SF-36 to measure QoL. In addition, transcriptome wide gene expression and 484 Single Nucleotide Polymorphysms (SNPs) in cytokine genes were available. QoL was first analyzed and associated with patient characteristics. Patients’ physical QoL was impaired and weakly related with age and scoliosis, whereas mental quality of life (MCS) was normal. To explain a largely lacking correlation between disease severity and QoL, we related genome wide gene expression to QoL. Patients with lower MCS scores had high expression levels of *CXCL9* and *CXCL11* cytokine-related genes (*p* = 0.001; *p* = 0.002); similarly, patients with low vitality scores had high expression levels of *CXCL9, CXCL11* and *IFNA6* cytokine-related genes (*p* = 0.02*; p* = 0.02; *p* = 0.04), independent of patient characteristics. Subsequently, we associated cytokine related SNPs to mental QoL (MCS and vitality). SNP-cluster in the *IL4R* gene showed a weak association with MCS and vitality (strongest association *p* = 0.0017). Although overall mental QoL was normal, >10% of patients had low scores for MCS and vitality. Post-hoc analysis of systemic inflammatory mediators showed that patients with lowest MCS and vitality scores had high levels of CCL11 cytokine (*p* = 0.03; *p* = 0.04).

**Conclusions/Significance:**

Variation in the cytokine genetic pathway and its activation is related to mental QoL. These findings might allow us to identify and, ultimately, treat patients susceptible to poor QoL.

## Introduction

Quality of life (QoL) is an emerging general parameter of patients’ well-being. QoL can be defined in various ways. It is a multifactorial concept consisting of individual perception of physical, psychological and social functioning [1]. In general, research has documented that patient characteristics, such as age, sex, racial, and psychological factors, such as mood states, and stress, influence patients' QoL [2]–[5]. However, there is a large variation between individuals that is not explained by these factors. This suggessts that intrinsic factors, i.e. individual genetic predisposition, contributes to ones perception of his or her well-being. In diverse psychiatric and psycological states a genetic disposition is emerging as an important causative factor. Such examples are negative emotional states (e.g. depression) [6], positive emotional states (e.g. subjective well-being) [7], self-rated health [8], pain [9] and fatigue [10]. In addition, results from twin studies show that the heritability for subjective well-being and life-satisfaction is up to 40–50%, whereas for both depression and anxiety this is around 30–40% [11]. Different biological pathways have been associated with various QoL elements, i.e. mood, overall well-being, pain and fatigue [12]. Examples of these biological pathways are the hypothalamic-pituitary-adrenal axis, immune, neuroendocrine, and cardiovascular systems [12].

The genetic basis for QoL has been largely ignored. Recently, the GENEQOL Consortium was initiated aiming to investigate the genetic disposition of QoL [13]. A first study by Rauch et al has shown that various single nucleotide polymorphisms (SNPs) in cytokine genes are related to QoL in lung cancer survivors [14].

In this study we investigated the QoL and its genetic basis in patients with Marfan syndrome (MFS). This is a chronic heritable disorder involving many organ systems at young age [15]. Main features are aortic root dilatation with risk of sudden death, skeletal deformities resuliting from overgrowth of long bones and dislocation of ocular lens. Most of the features require regular medical follow-up and often operative treatment at young age [16]. Living with MFS can have a profound impact on daily life [17] and one would expect impairments of patients’ QoL. Four studies have examined QoL in MFS patients [18]–[21]. Three of these studies reported impairments in physical QoL, whereas the mental QoL was similar compared to the normal population [18], [20], [21]. Only one study, however, explored the influence of disease characteristics on QoL and concluded that QoL cannot be explaned by disease severity in these patients. [20].

In this study we explored the QoL and the influece of disease severity on QoL in MFS patients. Furthermore, we searched for intrinsic factors which contribute to QoL by exploring gene expression, SNPs and cytokine levels in MFS patients.

**Table 1:** Patient characteristics in frequencies (percentages).

Patient characteristics	Total group(n = 121)	Non-responders (n = 107)
Demographics		
Age (mean, SD)	37 (13)	37 (12)
Sex (male)	67 (55)	53 (50)
Cardiac features		
Aortic root dilatation	100 (88)	90 (84)
Root replacement	41 (34)	35 (32)
Mitral valve prolapse	51 (42)	37 (32)
Beta-blocker usage*	92 (76)	65 (60)
Ectopia lentis*	58 (54)	37 (35)
Skeletal features		
Pectus deformity	86 (71)	72 (67)
Joint hypermobility	19 (17)	24 (22)
Severe scoliosis	23 (21)	29 (27)
Hindfoot deformity	54 (49)	33 (31)
Wrist and thumb sign	67 (55)	63 (59)
Striae	68 (61)	69 (64)
Pneumothorax	17 (15)	7 (7)

Note: Frequencies (percentages) for the presence of the symptoms are given for all dichotomous variables. Age is given in mean years (standard deviation).

* *p*<0.05.

## Methods

### Study Population and Procedure

The patient sample participating in the “COzaar in Marfan PAtients Reduces aortic Enlargement” (COMPARE) study [22], was included in this study. In short, the COMPARE study investigates the effect of losartan on aortic dilatation in patients with MFS. Inclusion criteria of the COMPARE study were: diagnosis of MFS according to the Ghent criteria and age ≥18 years. Exclusion criteria were: previous replacement of more than one part of the aorta, previous aortic dissection, angiotensin converting enzyme inhibitor or angiotensin receptor blocker usage, current pregnancy. The COMPARE study was conducted in four Marfan centers in the Netherlands (Academic Medical Center Amsterdam, Leiden University Medical Center, St. Radboud University Medical Center Nijmegen, Groningen University Medical Center). The study was approved by four medical ethics committees of the participating centers. All patients gave written and oral informed consent. Patients were in regular follow-up by a cardiologist. Each patient was seen by a research physician at inclusion to assess Marfan-related symptoms, disease history and medication use. Furthermore, a questionnaire measuring QoL was handed out by a research physician before consultation.

**Figure 1:**
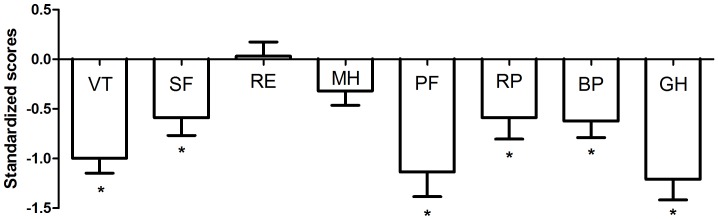
Standard quality of life scores on the eight quality of life domains. A standardized quality of life (QoL) score <0 indicates a QoL score that is worse than the age- and gender-matched reference population, and scores >0 indicates better QoL-scores. The standardized scores can be interpreted as Cohen’s d, indicating the effect size. VT = Vitality, SF = Social Functioning, RE = Role Emotional, MH = Mental Health, PF = Physical Functioning, RP = Role Physical, BP = Bodily Pain, GH = General Health. Domains which significantly (*p*<0.05) differed from the age- and gender-matched reference population are marked with *.

### Measurements

#### Patient characteristics

The following dichotomised (yes/no) MFS-related symptoms were assessed by research physicians at intake in the COMPARE study: aortic root dilatation, aortic root replacement, mitral valve prolapse, beta-blocker usage, pectus deformity, ectopia lentis, joint hypermobility (Beighton score >4/9), severe scoliosis (>20°), hindfoot deformity, wrist and thumb sign, striae, and pneumothorax. All features were scored according to the Ghent diagnostic criteria for MFS [23]. Aortic root dilatation and mitral valve prolapse were assessed by means of echocardiography at inclusion of the COMPARE study [22]. Additionally, age, sex, medication and co-morbidities were obtained at intake when QoL was measured.

**Table 2:** Patient characteristics independently related to quality of life.

Quality of Life	Patient characteristics	Explainedvariance (%)	
*MCS*	Severe scoliosis*	13.9	
	Age**		
Mental health	Striae	16.2	
	Bblockers*		
	Severe scoliosis**		
	Aortic root replacement		
	Age**		
Role Emotional	Ectopia lentis	8.6	
	Age*		
Social functioning	Striae	28.3	
	Bblockers*		
	Severe scoliosis**		
	Aortic root replacement		
	Age**		
Vitality	Severe scoliosis**	19.9	
	Sex*		
	Age**		
		
*PCS*	Severe scoliosis*	13.9	
	Age**		
General Health	Striae	20.5	
	Severe scoliosis**		
	Pneumothorax**		
	Age		
Bodily pain	Striae	13.1	
	Severe scoliosis*		
	Age*		
Physical function	Striae	20.2	
	Severe scoliosis		
	Sex		
	Age**		
Role physical	Aortic dilatation	20.0	
	Bblocker**		
	Aortic root replacement		
	Age**		

Note: All 121 patients were included. Presented are the independent variables wich were univariately significantly associated tih quality of life, and imultaneously included in multivariate linear regression analyses. SF-36 = Short Form MOS-36; MCS = Mental Component Summary; PCS = Physical Component Summary;

* *p<*0.05.

** *p<*0.01.

#### Quality of life

QoL was measured with the Short Form Health Survey-36 (SF-36), yielding eight domains, i.e. Vitality (VT; energy level), Social Functioning (SF; ability to participate in social activities), Role Emotional (RE; ability to participate in daily and occupation activities despite emotional constraints), Mental Health (MH; moods), Physical Functioning (PF; the ability to perform usual and energetic activities), Role Physical (RP; ability to participate in daily and occupation activities despite physical constraints), Bodily Pain (BP; pain level), and General Health (GH; current health) [24]. The first four domains load on a mental component summary (MCS), while the last four load on a physical component summary (PCS). For all eight domains and both subscales, higher scores reflect a better quality of life. The SF-36 is validated and yield good psychometric properties [25].

**Figure 2:**
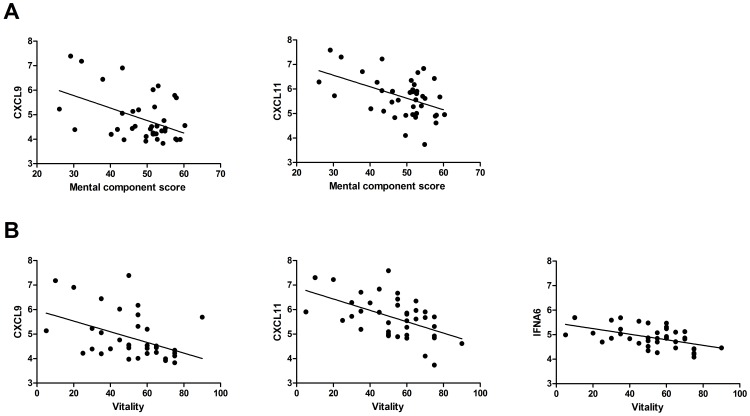
Scatterplots of gene expression levels of significantly associated genes (FDR = 0%) with mental quality of life. **A:** Scatterplot for the mental component score (*p*<0.002). **B:** Scatterplot for the vitality domain (*p*<0.04).

#### Gene expression

Genome wide expression, i.e. expression of all well referenced genes (approximately 18,000 genes) was measured in skin biopsies of patients using Affymetrix Human Exon 1.0 ST Array. Punch skin biopsies were performed in consenting patients after local anesthesia with ethyl chloride spray. Biopsies were immediately snap frozen in liquid nitrogen and stored at −80°C until further processing. Additional information about the RNA isolation and gene expression measurements is provided in the Supporting Information File, Text S1.

**Table 3:** Association of SNP clusters in *IL4R* gene with mental quality of life.

QoL^a^	SNP-ID	Chr^b^	Phys. Position^c^	Minor allele	Gene	*β* ^d^	*S.E.* ^ e^	*p* ^f^
**MCS** ^g^	rs4787423	16	27367334	C	*IL4R*	−4.5	1.5	0.0017
	rs3024536	16	27352713	T	*IL4R*	−3.6	1.7	0.0169
	rs3024537	16	27352819	A	*IL4R*	−3.6	1.7	0.0169
	rs3024544	16	27353357	T	*IL4R*	−3.6	1.7	0.0169
	rs3024570	16	27357784	A	*IL4R*	−4.1	2.0	0.0198
	rs3024633	16	27366499	A	*IL4R*	−4.1	2.0	0.0198
	rs4359426	16	57392733	A	*IL4R*	−5.2	2.8	0.0339
	rs170359	16	57395664	G	*IL4R*	−5.2	2.8	0.0339
	rs170360	16	57397950	C	*IL4R*	−5.2	2.9	0.0346
	rs3024560	16	27356667	G	*IL4R*	2.6	1.4	0.0363
	rs3024619	16	27364806	A	*IL4R*	2.5	1.4	0.0407
	rs2234895	16	27357927	T	*IL4R*	−3.5	2.1	0.0423
	rs3024607	16	27363611	A	*IL4R*	−3.5	2.1	0.0423
	rs6498011	16	27331894	A	*IL4R*	2.5	1.5	0.0466
	rs223819	16	57394862	C	*IL4R*	−4.3	2.7	0.0521

Note: a: QoL = Quality of life; b: Chr = chromosome; c: Phys. Position = Position of the SNP on the chromosome; d: *β = *mean MCS score difference per one minor allele; e: *S.E.* = standard error of the beta; f: *p* = p-value; g: MSC = Mental component score.

**Figure 3:**
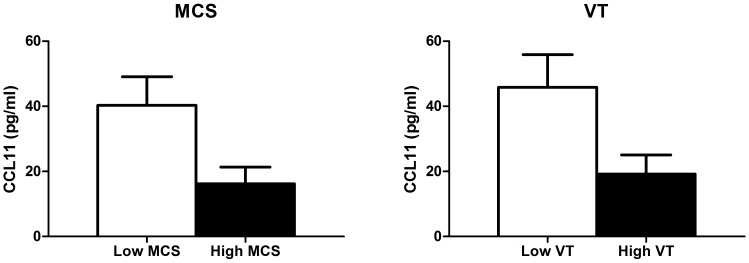
Differences in plasma levels of CCL11 between patients low and high on mental quality of life. Left: Differences in plasma levels of CCL11 between the 25% of patients with the lowest mental QoL (MCS) scores, and the 25% of patients with the highest MCS scores (*p* = 0.03). **Right:** Differences in plasma levels of CCL11 between patients with the lowest vitality (VT) score (standardized score ≤1.5) compared to the patients with high vitality scores (standardized score ≥0) (*p* = 0.04).

#### Single Nucleotide Polymorphisms (SNPs)

Genomic DNA was extracted from peripheral blood using the gentra puregene blood kit (Qiagen, the Netherlands) according to the manufacturer's instructions. Genotyping of the patients was performed using Illumina Human Omni Express Bead Chip measuring >700 000 SNPs. Multiple quality control measures were implemented. The estimated sex for each individual determined by genotyping was compared with their phenotypic sex. Exclusion criteria included deviation from Hardy-Weinberg equilibrium at *p*<10−3, sample call rate <0.95 and SNP call rate <0.98. Additional information about the SNP analysis is provided in the Supporting Information File, Text S1.

#### Cytokine measurements in plasma

Peripheral EDTA blood was sampled at the time of QoL measurement, centrifuged at 3220 rfm for 10 minutes, snap-frozen and stored in small portions at −80°C. Cytokines were measured using the suspension bead assays (Bio-Rad, Richmond, CA) using a Luminex reader (BioRad, BioSource, Linco). Lowest detection rate ranged from 0.12–0.91 pg/ml and intra- and inter-assay coefficients of variation <5%.

### Statistical Analyses

#### Quality of life

We first compared MFS patients’ QoL to the normal population. MFS patients’ mean scores on the eight QoL domains (see Table S1) were therefore transformed to standard scores based on the scores of an age- and gender-matched Dutch reference population [26]. Standard scores were calculated by dividing the difference between the mean scores of the MFS patients and the scores of the age- and gender-matched reference population, by the standard deviation of the reference population. The value of the standard scores can be interpreted according to Cohen’s effect size (*d*), where a score of <0.2 indicates a small, 0.5–0.8 a moderate and >0.8 a large difference. Additionally, the MCS and PCS scores were compared to the mean of the general population (mean scores = 50), by means of two t-tests at a significance level of 5%.

Second, the relation between patient characteristics and QoL was examined. As a first step, several linear regression analyses were employed to identify, which of the in Table 1 listed patient characteristics, were significantly (*p*<0.10) related to each of the QoL outcomes (i.e. the eight QoL-domains and both QoL-subscales). Subsequently, to examine the independent relation between patient characteristics and QoL, all univariately significant variables were simultaneously included in multivariate linear regression analyses. These analyses were performed for each of the QoL outcomes separately (*p*<0.05).

#### Relating gene expression and SNPs to quality of life

First, QoL-outcomes were correlated with the genome wide gene expression in skin using univariate linear regression analyses with permutation testing in Significance Analysis of Microarrays package in R program [27]. In order to avoid multiple testing problems, we chose a conservative False Discovery Rate (FDR) of 0% as significant. Subsequently, multivariate regression analyses were used to test whether the relation between gene expression and QoL remained significant after controlling for the significantly independently associated patient characteristics (*p*<0.05).

Second, SNPs belonging to the genes of the cytokine pathway found in the gene expression study (in the previous step) were selected for an association study. Only SNPs with a minor allele frequency >5% were analyzed. Linear regression analysis assuming an additive genetic model with adjustment for age and sex was used. For all analyses we used the GenABEL analysis package in R program [28]. Bonferroni correction for multiple testing was used to define the target p-value.

## Results

### Patient Characteristics and their Relation with Quality of Life

Of the 228 selected patients from the COMPARE study [22], 121 patients filled out the SF-36 (response rate 52.6%) and were thus included in this study. Patient characteristics for the patient group included in this study (n = 121) and non-responders (n = 107) are presented in Table 1. Only ectopia lentis and usage of beta-blockers were more frequent in the investigated group (n = 121) compared to the non-responders, Table 1. Gene expression was available for 40 of the 121 patients (33%), whereas genotypes (SNPs) were available for 111 patients. Patient characteristics (Table 1) of both groups were compared to the total group (n = 121). Results showed that ectopia lentis and two skeletal features (severe scoliosis and hindfoot deformity) were more frequent in the total group (n = 121) compared to the group for whom gene expression was available (n = 40).

When QoL of MFS patients (n = 121) was compared to that of an age- and gender-matched population, scores on six of the eight QoL domains were significantly lower in MFS patients (*p*<0.01) (Figure 1). MFS patients had a similar mental QoL (*t* (116) = −0.5, *p = *0.63) as the general population. The physical QoL of MFS patients was significantly lower than that of the age- and gender-matched population (*t*(116) = −4.8, *p*<0.01). On average 10% of patients had an impaired QoL (standardized score ≤2, data not shown).

Results of the analyses relating patient characteristics to QoL showed that correlations were largely lacking, only age and the presence of severe scoliosis were independently related to the QoL-domains and -subscales (*p*<0.05). The variance in QoL explained by these characteristics ranged from 8.9% to 28.3%, see Table 2.

### Expression of Inflammatory Genes are Independently Related to Variablity in Mental Quality of Life

Since correlations between patient characteristics and QoL were weak and the explained variance was low, we explored whether variation in QoL was related to gene expression in MFS patients (n = 40). Results of these analyses revealed that physical QoL was not correlated with gene expression. Mental QoL (MCS and vitality), however, was associated with expression of genes coding for cytokines. MCS was associated with the genes *CXCL9* and *CXCL11* (*FDR* = 0% for all; *t*(39) = −2.9; *t*(39) = −2.8 respectively). Vitality was associated with expression levels of *CXCL9*, *CXCL11* and *IFNA6* genes (*FDR* = 0% for all; *t*(39) = −3.2; *t*(39) = −2.6; *t*(39) = −2.6, respectively). Thus, patients with a worse mental QoL have more active cytokine genes (Figure 2).

This relation between gene expression and mental QoL and vitality remained significant, after controlling for the significant independent related patient characteristics reported in Table 2. Mental QoL (i.e. MSC) was related to the genes *CXCL9* (*β* = −3.6, *p* = 0.001) and *CXLC11* (*β* = −3.4, *p* = 0.002), after controlling for age and the presence of severe scoliosis, which were both no longer significantly related to QoL. The explained variance in mental QoL increased from 13.9% (only patient characteristics) to an average of 35% (patient characteristics and gene expression). Vitality remained significantly associated with expression levels of, *CXCL9* (*β* = −2.5, *p* = 0.02), *CXCL11* (*β* = −2.5, *p* = 0.02), and *IFNA6* (*β* = −2.2, *p* = 0.04) after controlling for patient characteristics (i.e. age, sex, and the severe scoliosis). Only age remained an independent significant correlate with vitality. The explained variance in vitality scores increased from 19.9% to an average of 42%. Given the immunological character of the associated genes, we explored the prevalence of immunological co-morbidities and clinical depression. Both prevalence levels were comparable to the general population (Table S2); there were no significant differences.

### SNPs in *IL4R* Gene Correlate with Mental Quality of Life

As mental QoL was associated with the expression of cytokine genes, we selected SNPs in 88 cytokine-related genes and 2 kb up- and down-stream of these (See Table S3 for the full list). In total 659 SNPs were found in these genes of which 484 passed the quality control and were included in the analysis. Results of the association study of the 484 SNPs showed that the mental QoL (i.e. MCS) and vitality were associated with several SNPs with the highest *p*-value of 0.0017 (Table S4). MCS was associated with a top SNP rs4787423 (*p = *0.0017) located in the *IL4R* gene (Table 3). Several SNPs in the *IL4R* gene had a significant p-value forming a trail (Figure S1). Vitality showed the strongest association in rs2023906 SNP (*p* = 0.0017), single SNP in *CCR7* gene which suggests this finding is likely to be incidental.

Although mental QoL and vitality scores were not related to MFS symptoms, there was an overall trend to lower mental QoL scores with approximately 10% of patients having low mental QoL scores. Therefore we post-hoc explored the systemic levels of inflammatory markers (i.e. IL4, IL5, IL13, GM-CSF and CCL11) in patients with the lowest mental QoL and vitality scores. These cytokines are in the same inflammatory pathway as the *IL4R* gene and are all involved in the pathogenesis of allergy (e.g. asthma and rhinitis). The 25% of patients with the lowest MCS scores were compared to the 25% patients with the highest mental QoL. In this line, patients with the lowest vitality scores (standardized score ≤1.5) were compared to patients with normal scores (standardized score ≥0). Results from both comparisons showed that patients with a low mental QoL had higher levels of CCL11 in plasma blood compared to patients with normal mental QoL (*p* = 0.03 and *p* = 0.04), Figure 3.

## Discussion

In our study, physical QoL of MFS patients was impaired and related to age and presence of scoliosis. Mental QoL, however, was comparable to the normal population. Both mental QoL and vitality, one of its components, were associated with genetic variation in cytokine genes and their activity, independently of patient characteristics (i.e. demographics, MFS-related symptoms and co-morbidities). Moreover, the impairments in QoL were not associated with MFS-related genes (i.e. genes in the TGF-β pathway). Therefore, this relation seems to be not MFS specific and might be extrapolated to the normal population. In a small subgroup of patients with impaired mental QoL and vitality high systemic levels of the cytokine CCL11 were found.

Our primary finding, that the variation in mental QoL can be independently explained by cytokine-related genes, is in concordance with the emerging literature about the immunological basis of QoL-related symptoms and disorders such as depression and fatigue [29]–[35]. It is important to note that although related, depression, fatigue and QoL are distinct concepts. In our study both mental QoL and vitality were independently, negatively related to *CXCL9* and *CXCL11* expression levels. These cytokines belong to the same family together with *CXCL10* and share the common receptor *CXCR3*. In normal conditions their levels are generally not detectable but are strongly induced by interferon-gamma (IFN- γ) [36]. Previous research has demonstrated that global QoL was negatively related to IFN-γ [37]. Serum levels of the related *CXCL10* gene were found to be related to depression [38], [39]. Additionally, in our patient population vitality was negatively related to the *IFNA6* gene. Although there is little known about the function of this gene, IFN-induced fatigue is a common phenomenon [31], [32].

Interestingly, the SNPs in *CXCL9, CXCL10, CXCL11* and *IFN* genes were not significantly associated with QoL. High expression levels of these genes are most likely induced by other inflammatory genes up-stream in a complex immunological cascade. In line with this hypothesis, we found a relation between mental QoL and SNPs in the *IL4R* gene. This gene codes for the IL4 receptor. Polymorphisms in this gene are associated with asthma and rhinitis in case-control studies [40], [41]. In our patient population the prevalence of immunological and allergic disorders was not increased suggesting SNPs in the *IL4R* gene independently contributed to mental QoL. Numerous studies have shown that higher IL4 levels are protective for depression [30], [42], [43]. Mechanisms through which inflammatory genes and QoL are related are unknown. Hypothetically, high expression levels of cytokine-related genes are associated with sickness-behavior which is reflected in impaired QoL. In our previous work we found that inflammation aggravates disease severity in MFS patients. In this study however, mental QoL was associated with cytokine related genes independently of disease characteristics, suggesting an independent immune-related process which affects the perception of the well-being in these patients. Functional immunological studies are needed in order to elucidate the exact mechanism of this relation.

Although mental QoL and vitality were unrelated to MFS, there was a high number of patients, approximately 10%, with low scores for these two components of mental QoL. These patients showed high systemic levels of the CCL11, a cytokine involved in the pathogenesis of allergy. Similar results have been found in depressed patients; they showed elevated CCL11 levels compared to age- and gender-matched controls [44]. In this study only clinically treated depression was documented. We cannot exclude the possibility that some patients with low QoL have depressive symptoms. Other factors which were not measured in this study could also contribute to QoL impairments. For example, MFS patients are likely to have emotional problems, such as feelings of guilt because of inheritance of MFS and concerns about their career, family planning, insurance and housing [45]. In addition, factors not related to MFS, such as lack of social support and personality may play a role as well. Future studies should explore additional factors associated with QoL in MFS patients.

There are some limitations to this study. First, most severe cardiovascular MFS patients were not included, due to the exclusion criteria of the COMPARE study. This might have led to an underestimation of the impairment in physical QoL and its relation with cardiovascular disease severity. The main limitation of this study is a relatively small sample size. The highest p-value (*p* = 0.0017) in the genetic association study did not reach the targeted p-value after Bonferroni correction (*p* = 0.0001). Most SNPs in the *IL4R* gene had p-values less than 0.05 which suggest the association is not likely to be incidental. As these associations do not seem to be MFS specific, validation in patients with different pathology or even in a healthy population could yield interesting results. This is still the largest study on QoL in MFS patients and the first study exploring the genetic basis of their QoL.

In conclusion, we found a genetic basis for mental QoL in cytokine genes and their activity. This relation does not seem specific for MFS and is independent of patient characteristics. Knowledge about this genetic component of QoL provides insight and can eventually allow us to identify patients susceptible to poor QoL. This information might guide clinicians in deciding making, opting for treatments with the smallest negative impact on QoL. Furthermore, we will be able to better target specific support to those who need it. Note that validation in larger patient populations is warranted. Ultimately, immunological treatment strategies can be developed to improve patients’ QoL.

## Supporting Information

Figure S1 Locus-specific association map generated from genotyped SNPs in IL4R gene, centered at rs4787423.
(DOC)

Table S1 MFS patients’ mean scores on the quality of life domains and subscales.
(DOC)

Table S2 Prevalence of immunologically-related disorders and treated depression in our MFS population.
(DOC)

Table S3 Included SNPs.
(XLS)

Table S4 Top SNPs associated with quality of life.
(DOC)

Text S1 Contains detailed information about the gene expression study and genotyping.
(DOC)
