# Supplementary material for: Mental Quality of Life Is Related to a Cytokine Genetic Pathway
Source: PLoS One. 2012 Sep 25;7(9):e45126. doi: 10.1371/journal.pone.0045126 (PMC3458023; doi:10.1371/journal.pone.0045126)
Supplement: Figure S1 — Locus-specific association map generated from genotyped SNPs in IL4R gene, centered at rs4787423. (DOC) [file pone.0045126.s001.doc]

**FIGURES**

*Figure S1:* Locus-specific association map generated from genotyped SNPs in *IL4R* gene, centered at rs4787423.
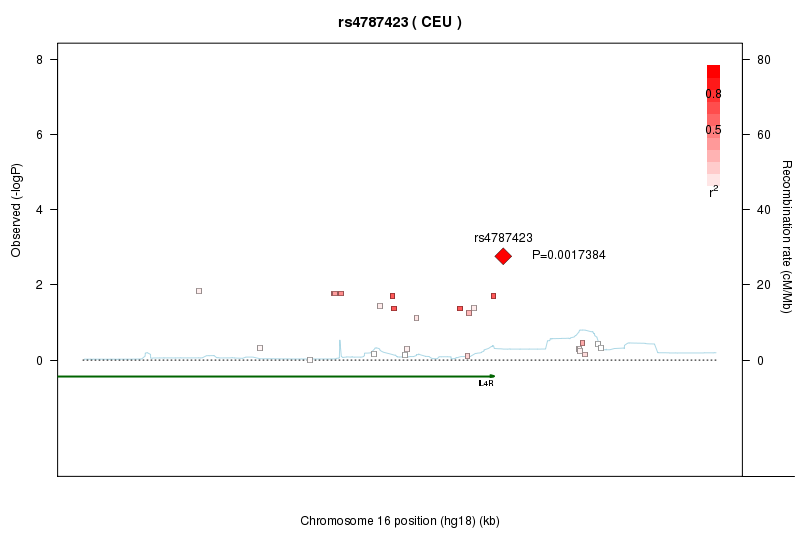


Legend Figure 1: SNPs in red have r2 ≥ 0.8 with rs2824293; SNPs in orange have r2=0.5–0.8; SNPs in yellow have r^2^=0.2–0.5; and SNPs in white have r^2^ < 0.2 with the leading SNP. Superimposed on the plot are gene locations (green) and recombination rates (blue). Chromosome positions are based on HapMap release 22 build 36.2 and b was prepared using SNAP.^ref^

**Reference**

Johnson AD, Handsaker RE, Pulit SL, Nizzari MM, O'Donnell CJ, et al. SNAP: a web-based

tool for identification and annotation of proxy SNPs using HapMap. Bioinformatics

2008;24(24):2938-2939.
