# Supplementary material for: Mental Quality of Life Is Related to a Cytokine Genetic Pathway
Source: PLoS One. 2012 Sep 25;7(9):e45126. doi: 10.1371/journal.pone.0045126 (PMC3458023; doi:10.1371/journal.pone.0045126)
Supplement: Table S1 — MFS patients’ mean scores on the quality of life domains and subscales. (DOC) [file pone.0045126.s002.doc]

*Table S1:* Mean scores on the quality of life domains and subscales

| **Quality of life** | **Mean (SD)** |
| --- | --- |
| *MCS* | 49.59 (9.09) |
| Mental health | 75.59 (16.28) |
| Role emotional | 84.31 (30.94) |
| Social functioning | 79.58 (21.80) |
| Vitality | 58.77 (19.43) |
| *PCS* | 45.46 (10.32) |
| General health | 56.99 (22.46) |
| Bodily pain | 70.58 (23.80) |
| Physical functioning | 79.23 (20.25) |
| Role physical | 68.80 (41.37) |

SD= standard deviation; MCS = Mental Component Summary; PCS = Physical Component Summary
