# Supplementary material for: Mental Quality of Life Is Related to a Cytokine Genetic Pathway
Source: PLoS One. 2012 Sep 25;7(9):e45126. doi: 10.1371/journal.pone.0045126 (PMC3458023; doi:10.1371/journal.pone.0045126)
Supplement: Table S2 — Prevalence of immunologically-related disorders and treated depression in our MFS population. (DOC) [file pone.0045126.s003.doc]

*Table S2:* Prevalence of immunologically-related disorders and treated depression in MFS population

| **Disorder** | **Prevalence study population (%)** | **Prevalence Dutch population (%)** |
| --- | --- | --- |
| Rheumatoid arthritis | 0.8 | 0.5 |
| Asthma | 3.3 | 2.5 |
| Allergic rhinitis | 0.8 | 15 |
| Depression | 5.5 | 4.2 |

Numbers are provided in percentages
