# Supplementary material for: Mental Quality of Life Is Related to a Cytokine Genetic Pathway
Source: PLoS One. 2012 Sep 25;7(9):e45126. doi: 10.1371/journal.pone.0045126 (PMC3458023; doi:10.1371/journal.pone.0045126)
Supplement: Table S4 — Top SNPs associated with quality of life. (DOC) [file pone.0045126.s005.doc]

*Table S4:* Top SNPs associated with quality of life

| **QoL** | **SNP-ID** | **Chr** | **Position** | **Min. allele** | **Gene** | ***β*** | **S.E.** | ***p*** |
| --- | --- | --- | --- | --- | --- | --- | --- | --- |
| **MCS** | rs4787423 | 16 | 27367334 | C | *IL4R* | -4.5 | 1.5 | 0.0017 |
|  | rs2546893 | 5 | 158755960 | A | IL12B | 3.9 | 1.4 | 0.0026 |
|  | rs2302004 | 7 | 75442855 | A | CCL24 | 3.5 | 1.3 | 0.0032 |
|  | rs3136667 | 3 | 46244284 | G | CCR1 | -5.8 | 2.1 | 0.0032 |
|  | rs11741953 | 5 | 55267814 | C | IL6ST | -6.0 | 2.2 | 0.0036 |
|  | rs10471960 | 5 | 55236374 | G | IL6ST | -5.6 | 2.2 | 0.0062 |
|  | rs4732416 | 7 | 75441762 | C | CCL24 | 3.4 | 1.4 | 0.0068 |
|  | rs355667 | 4 | 78495883 | C | CXCL13 | 3.2 | 1.4 | 0.0100 |
|  | rs13073976 | 3 | 46286663 | C | CCR1 | -5.5 | 2.4 | 0.0117 |
|  | rs4987053 | 3 | 46306700 | T | CCR1 | -5.5 | 2.4 | 0.0117 |
|  | rs2193873 | 3 | 190313507 | A | IL1RAP | 3.0 | 1.4 | 0.0141 |
|  | rs4787948 | 16 | 27341059 | G | *IL4R* | 3.3 | 1.5 | 0.0147 |
|  | rs7650510 | 3 | 190363116 | G | IL1RAP | -3.4 | 1.6 | 0.0148 |
|  | rs2046068 | 12 | 68645975 | G | IL22 | 3.6 | 1.6 | 0.0153 |
|  | rs3024536 | 16 | 27352713 | T | *IL4R* | -3.6 | 1.7 | 0.0169 |
|  | rs3024537 | 16 | 27352819 | A | *IL4R* | -3.6 | 1.7 | 0.0169 |
|  | rs3024544 | 16 | 27353357 | T | *IL4R* | -3.6 | 1.7 | 0.0169 |
|  | rs2856762 | 3 | 46413334 | T | CCR5 | -4.5 | 2.2 | 0.0188 |
|  | rs2227491 | 12 | 68646521 | T | IL22 | 3.3 | 1.6 | 0.0198 |
|  | rs3024570 | 16 | 27357784 | A | *IL4R* | -4.1 | 2.0 | 0.0198 |
|  | rs3024633 | 16 | 27366499 | A | *IL4R* | -4.1 | 2.0 | 0.0198 |
|  | rs14304 | 17 | 34398495 | T | CCL18 | -2.9 | 1.5 | 0.0233 |
|  | rs11080369 | 17 | 34305164 | C | CCL18 | -5.1 | 2.7 | 0.0271 |
|  | rs976881 | 1 | 12233754 | T | TNFRSF1B | -2.6 | 1.4 | 0.0302 |
|  | rs4149576 | 12 | 6449115 | T | TNFRSF1A | -2.5 | 1.3 | 0.0308 |
|  | rs4359426 | 16 | 57392733 | A | CCL22 | -5.2 | 2.8 | 0.0339 |
|  | rs170359 | 16 | 57395664 | G | CCL22 | -5.2 | 2.8 | 0.0339 |
|  | rs189587 | 4 | 78500085 | A | CXCL13 | 2.6 | 1.4 | 0.0340 |
|  | rs170360 | 16 | 57397950 | C | CCL22 | -5.2 | 2.9 | 0.0346 |
|  | rs11465307 | 7 | 75441794 | G | CCL24 | 3.1 | 1.7 | 0.0352 |
|  | rs1800693 | 12 | 6440009 | C | TNFRSF1A | -2.5 | 1.4 | 0.0353 |
|  | rs3024560 | 16 | 27356667 | G | *IL4R* | 2.6 | 1.4 | 0.0363 |
|  | rs6861772 | 5 | 55271621 | G | IL6ST | -4.1 | 2.3 | 0.0372 |
|  | rs9831803 | 3 | 190361497 | T | IL1RAP | -3.3 | 1.8 | 0.0381 |
|  | rs767455 | 12 | 6450945 | C | TNFRSF1A | -2.4 | 1.4 | 0.0394 |
|  | rs3024619 | 16 | 27364806 | A | *IL4R* | 2.5 | 1.4 | 0.0407 |
|  | rs2234895 | 16 | 27357927 | T | *IL4R* | -3.5 | 2.1 | 0.0423 |
|  | rs3024607 | 16 | 27363611 | A | *IL4R* | -3.5 | 2.1 | 0.0423 |
|  | rs12719919 | 10 | 6054158 | T | IL2RA | -4.6 | 2.7 | 0.0436 |
|  | rs6498011 | 16 | 27331894 | A | *IL4R* | 2.5 | 1.5 | 0.0466 |
|  | rs223819 | 16 | 57394862 | C | CCL22 | -4.3 | 2.7 | 0.0521 |
|  | rs10489630 | 1 | 67662622 | C | IL23R | -2.3 | 1.4 | 0.0524 |
|  | rs3024614 | 16 | 27364345 | A | IL4R | -4.83 | 3.0 | 0.055 |
| **VT** | rs2023906 | 17 | 38713686 | G | CCR7 | -8.5 | 2.9 | 0.0017 |
|  | rs12551256 | 9 | 6231239 | G | IL33 | 6.9 | 2.7 | 0.0050 |
|  | rs12719919 | 10 | 6054158 | T | IL2RA | -12.0 | 5.3 | 0.0124 |
|  | rs2046068 | 12 | 68645975 | G | IL22 | 7.5 | 3.4 | 0.0146 |
|  | rs16924159 | 9 | 6229417 | A | IL33 | 6.2 | 3.1 | 0.0247 |
|  | rs1126580 | 2 | 219000966 | A | IL8RB | -6.1 | 3.2 | 0.0275 |
|  | rs12510514 | 4 | 142601496 | A | IL15 | -7.9 | 4.1 | 0.0283 |
|  | rs10833 | 4 | 142654547 | C | IL15 | -6.2 | 3.3 | 0.0291 |
|  | rs9849030 | 3 | 190257179 | G | IL1RAP | 7.9 | 4.3 | 0.0332 |
|  | rs2227491 | 12 | 68646521 | T | IL22 | 6.3 | 3.5 | 0.0355 |
|  | rs13122930 | 4 | 142650413 | A | IL15 | 5.7 | 3.2 | 0.0358 |
|  | rs6850492 | 4 | 142637305 | A | IL15 | -5.8 | 3.2 | 0.0364 |
|  | rs1330383 | 9 | 6251507 | T | IL33 | 5.3 | 3.0 | 0.0369 |
|  | rs16924144 | 9 | 6221246 | C | IL33 | 5.5 | 3.1 | 0.0374 |
|  | rs10975516 | 9 | 6247693 | A | IL33 | 5.3 | 3.0 | 0.0384 |
|  | rs1519551 | 4 | 142570472 | G | IL15 | -5.1 | 2.9 | 0.0391 |
|  | rs4251985 | 2 | 113877413 | T | IL1RN | -5.8 | 3.5 | 0.0455 |
|  | rs2322301 | 4 | 142636313 | C | IL15 | 5.5 | 3.3 | 0.0456 |
|  | rs12244380 | 10 | 6053374 | G | IL2RA | -4.9 | 2.9 | 0.0479 |
|  | rs10975519 | 9 | 6253571 | T | IL33 | 5.0 | 3.0 | 0.0502 |
|  | rs3024570 | 16 | 27357784 | A | IL4R | -7.3 | 4.5 | 0.0507 |
|  | rs3024633 | 16 | 27366499 | G | IL4R | -7.3 | 4.5 | 0.0507 |

Note: The sample size in these analyses was 111 patients. QoL = quality of life; MCS = Mental Component Summary; VT = vitality; Chr = chromosome; Min. Allele = minor allele; *S.E.* = standard error of the beta; *β* = mean MCS score difference per one minor allele; *p* = p-value
