# Supplementary material for: Mental Quality of Life Is Related to a Cytokine Genetic Pathway
Source: PLoS One. 2012 Sep 25;7(9):e45126. doi: 10.1371/journal.pone.0045126 (PMC3458023; doi:10.1371/journal.pone.0045126)
Supplement: Text S1 — Contains detailed information about the gene expression study and genotyping. (DOC) [file pone.0045126.s006.doc]

**METHODS**

**Gene expression study**

Full skin punch biopsies of 4 mm (~5-15 mg) were used to isolate total RNA. Skin biopsies were pulverized in liquid nitrogen and transferred to 1.5 ml tubes containing Qiazol (Qiagen). Crude RNA extractions were obtained according to manufacturer’s instructions with the addition of Phase-Lock Gel Heavy (5 Prime) to obtain a better phase separation. The crude RNA fractions were further purified with the RNeasy Minelute Cleanup Kit (Qiagen) according to Appendix D protocol: RNA Cleanup after Lysis and Homogenization with QIAzol Lysis Reagent. RNA yield was measured on a Nanodrop ND-1000 (Thermo Fisher Scientific) and the RNA quality was investigated on the BioAnalyzer 2100 (Agilent Technologies) with the RNA 6000 Pico Chip Kit (Agilent Technologies). Only RNA samples with sufficient yield and RIN-values above 6.5 were used for analysis.

Gene expression was analyzed with Affymetric Human Exon 1.0 ST Arrays. Sense-strand cDNA was generated from total RNA using Ambion WT Expression Kit (Applied Biosystems) conform manufacturer’s instructions. Further steps were performed using manufacturer’s protocols for the GeneChip platform (Affymetrix). Those included purification of double-stranded cDNA, synthesis of cRNA by in vitro transcription, recovery and quantitation of biotin-labeled cRNA, fragmentation of this cRNA and subsequent hybridization to the microarray slide, posthybridization washings and detection of the hybridized cRNA using a streptavidin-coupled fluorescent dye. Hybridized Affymetrix Arrays were scanned using Gene-Chip Scanner 3000-7G (Affymetrix). Image generation and feature extraction were performed using Affymetrix GCOS Software v1.4.0.036.

Normalization and quality control were performed using the Expression Console © software by Affymetrix.

**Genotyping**

Genomic DNA of MFS patients was extracted from peripheral blood using the gentra puregene blood kit (Qiagen, the Netherlands) according to the manufacturer's instructions. Microarray-based DNA genotyping was performed at ServiceXS (ServiceXS B.V., Leiden, The Netherlands) using the HumanOmniExpress BeadChip (Illumina, Inc., San Diego, CA, U.S.A). This array interrogates >700,000 loci with a median marker spacing of 2.2 kb. Of each sample, 4 µl genomic DNA at 50 ng/µl was processed and hybridized to the BeadChips, according to the manufacturer's instructions. The BeadChip images were scanned on the iScan system and the data was extracted into Illumina's GenomeStudio software v2010.1. The software's default settings were used with the cluster file as developed by Illumina for genotype calling.

Statistical analysis was performed using GenABEL package^ref^ in the R statistical program (R Development Core Team (2010). R: A language and environment for statistical computing. R Foundation for Statistical Computing, Vienna, Austria. ISBN 3-900051-07-0, URL http://www.R-project.org). Linear regression analysis assuming an additive genetic model with adjustment for age and sex was used to analyze the association of the aortic root dilatation rates and genotypes. Multiple quality control measures were implemented. The estimated sex for each individual determined by genotyping was compared with their phenotypic sex. Only SNPs with a minor allele frequency of >5% were included in the analysis. Exclusion criteria included deviation from Hardy-Weinberg equilibrium at p<10−3, sample call rate <0.95 and SNP call rate <0.98. Bonferroni correction for the target p-value was applied.

**Reference**

Aulchenko YS, Ripke S, Isaacs A, van Duijn CM. GenABEL: an R library for genome-wide association analysis. Bioinformatics 2007;23(10):1294-1296.
